# Cleaning Pennsylvania’s Mouth

**Published:** 1923-06

**Authors:** 


### CLEANING PENNSYLVANIA'S MOUTH

Not that Pennsylvania's mouth is more in need of cleaning than that of any other state—the mouth of America as a whole is a health menace, so far as the state of its teeth is concerned—but that this being the second most populous state in the Union, naturally the effort to make it sounder and more efficient assumes a place of large importance among welfare movements. And this is what the state department of health now has started out to do.

Of course, the place to begin is with the children, and that is where the start is being made. Seven or eight years ago the late Dr. Samuel G. Dixon, then commissioner of health, sounded the first note in this battle against bad teeth by instructing school doctors to include teeth in their annual physical examination of the boys and girls.

At that time the average physician knew little about the intimate relation of good teeth to good health, and the average dentist was not yet aware of the vast amount of disease and suffering due primarily to mouth centers of infection. Discoveries along these lines have come thick and fast within the last five years, and these revelations have led to a new spirit of co-operation between dentists and physicians. In the current issue of the *Journal of the American Medical Association,* for instance, an eminent medical authority says: "There is no more interesting or profitable field of collateral study for the physician than that of the problems of the teeth, as portrayed by modern dentistry, and I believe dentists will find equal satisfaction in a survey of the changes in the body resulting from disease, the cause of which often finds expression in changes in the teeth."

To lay a broad and firm foundation for such co-operative work in behalf of public health is the underlying aim of the dental campaign now in progress. Deeply impressed with the sound principles on which school dental prophylaxis is based and the sane methods modern science has devised for applying these principles, the state department of health has determined to further this work in every way possible.

From the start it was seen that mere lessons in mouth hygiene would not suffice, for more than ninety per cent. of the mouths of all school children in this state today are in a condition which demands something beyond preventive knowledge. Corrective treatment is called for in these cases, yet it can easily be seen that this implies a task to be accomplished only by the solidest kind of united public support. It also is evident to any thoughtful person that to make this work most effective the first move should be directed against the foe in the mouths of young children.

Early last year a motor dental ambulance was made available to the department and a dental unit was formed, consisting of a dentist and two mouth hygienists. Up to that time prophylactic dental service had not been attempted in any of the schools in this state, though school dental clinics had been established in various sections.

School boards of certain cities were asked to consent to a demonstration in their schools, consisting of actual cleaning and polishing of the children's teeth, a charted examination of every mouth, toothbrush drills and classroom talks on dental hygiene, and filling of small cavities. Each of these demonstrations occupied from ten days to two weeks and the work was confined to fourth-grade pupils.

During a period of four months twelve communities, varying in size from 12,000 to 150,000, were visited. In this preparatory campaign 644 pupils were examined, and it was found that 93 per cent. of these had unclean teeth and 96 per cent. decayed teeth. The total number of cavities found in these 644 mouths was 3933, an average of more than six to each mouth. More than 71 per cent. of the children examined used a toothbrush at such irregular or infrequent intervals as to be valueless, or did not use one at all. And the chief of the division of school health said in his report that a great majority of the 29 per cent. who cleaned their teeth daily did not use efficient methods.

This demonstration proved conclusively to the communities visited that the mouths of school children present a problem that calls for prompt state-wide action. So impressed was one school board with the situation as revealed that it immediately voted the sum of $5,800 for dental prophylaxis during the present school year. Similar action on a smaller scale was taken by several other boards. And the successful results of this trial determined Commissioner Martin to push this work and make it an integral part of school health service throughout the state.

That this most important work may be properly carried on, a dental division of the health department has been created as a separate activity, and Dr. C. J. Hollister, a well-known dental surgeon of this city, appointed its chief. With a completely equipped motorized dental laboratory and an assistant hygienist, he now is visiting schools in different parts of the state to lay the foundation for this movement to make Pennsylvania's mouth a health portal rather than an open door to disease. And to provide for the support and necessary enlargement of this movement there is before the present legislature a bill which, if passed, will give this state perhaps the most proficient service in this line in the country.

It is, of course, gratifying to "The North American" that this necessary movement now is well under way. For ten years this newspaper has been urging upon every health agency and the people in general the need for proper care of the teeth. Within that period it has become an obvious fact that good teeth are a necessary adjunct to health, and public health is the very foundation of public welfare and all worth-while progress. It has been proved beyond doubt that numerous affections which once were laid to other causes or accepted as mysterious manifestations of unknown origin are due to so-called focal centers of infection that can not exist in a clean mouth of sound teeth.

Some idea of the extent of this relationship between diseases of the mouth and general disease may be gained from the following brief resume, written by the authority above quoted:

The degenerative diseases, including arteriosclerosis and chronic nephritis, appear to depend for their cause in part on chronic infections derived from the mouth. In a study of the teeth of 329 patients with various diseases in Cook County Hospital, of whom 124 were roentgenographed, alveolar abscesses were found in 44 per cent. When these patients were classified according to disease, there were found 76 per cent. of alveolar abscesses in the arthritic group, 47 per cent. of abscesses in the cardiovascular group, and in the remainder, including acute diseases, such as typhoid fever and pneumonia, 23 per cent. of abscesses.

Other investigators have found a still higher percentage of alveolar abscesses in various groups, and have also emphasized the effect of chronic infection in the production of chronic degenerative diseases.

In roentgenograms of 600 adults, Black found 78 per cent. showing areas of bone destruction; but in 72 per cent. there were as yet no evidences of systemic disease. More difficult of demonstration, but of apparently none the less importance to the general health, is the direct absorption of infection and the swallowing of pus derived from infected gums of pyorrhea. The absence of immediate symptoms indicates not that progressive damage is not occurring, but only that as yet the effects have not become clinically visible.

To combat this condition, which is causing so much suffering and expense and so seriously interfering with economic efficiency, all we need is general knowledge and use of the toothbrush. Of course, such a measure will not repair decayed teeth or undo damage already done. Even in such cases it will help, however, and if firmly planted among the children of the nation it will prove a genuine safeguard against continuance of present-day lamentable conditions.

We hope the people of Pennsylvania are wise and thrifty enough to see the need for hearty co-operation with the department of health in this matter of tooth training. For, of course, the success of the effort depends finally upon the support it receives in the homes.—*The North American.*

